# Metabolite to Modifier: Lactate and Lactylation in the Evolution of Tumors

**DOI:** 10.1002/mco2.70413

**Published:** 2025-10-05

**Authors:** Long Zhao, Haoyue Cui, Yutong Li, Yingjiang Ye, Zhanlong Shen

**Affiliations:** ^1^ Department of Gastroenterological Surgery Peking University People's Hospital Beijing China; ^2^ Laboratory of Surgical Oncology Peking University People's Hospital Beijing China; ^3^ Department of Cancer Biology Dana‐Farber Cancer Institute Boston Massachusetts USA; ^4^ Department of Biological Chemistry and Molecular Pharmacology Harvard Medical School Boston Massachusetts USA

**Keywords:** cancer therapy, immune modulation, lactate, lactylation, metabolic reprogramming, tumor microenvironment

## Abstract

Lactate, once dismissed as a mere by‐product of cancer metabolism, has emerged as a pivotal factor in tumor progression, exerting diverse effects on metabolic reprogramming and immune modulation. Lactate enhances tumor cell adaptability through sustained glycolysis and concurrently shapes the tumor microenvironment by modulating immune, stromal, and endothelial cell function. This review highlights the evolving understanding of lactate's role, extending beyond the Warburg effect to its regulatory capacity via lactylation, a recently identified post‐translational modification. The complex interaction between lactate and tumor biology is examined, emphasizing its influence on the tumor microenvironment and immune dynamics. Additionally, potential therapeutic strategies targeting lactate metabolism and transport are explored, along with lactylation regulation by histone‐modifying enzymes. Inhibitors targeting lactate production and transport, especially those against lactate dehydrogenase (LDH) and monocarboxylate transporters (MCTs), have shown considerable potential in preclinical and early clinical studies. Recent advancements are discussed, underscoring the potential of integrating metabolic regulation with immunotherapies, thereby offering a dual pathway in cancer treatment. These insights establish lactate and lactylation as pivotal modulators of tumor biology and highlight their potential as targets in precision oncology.

## Graphical abstract

Tumor cells produce a significant amount of lactate due to the Warburg effect, leading to an increase in extracellular lactate, which inhibits the function of immune cells. Concurrently, the intracellular lactate accumulation results in enhanced lactylation of proteins, predominantly histones, thereby promoting the initiation and progression of cancer.

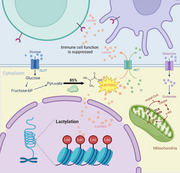

## 1 Introduction

The metabolic peculiarities of cancer cells, epitomized by the Warburg effect, have long captivated scientific inquiry. Since Otto Warburg's pioneering observations in the 1920s, lactate, once regarded as a by‐product of anaerobic metabolism, has been redefined as a key player in the metabolic framework of tumors [1]. This paradigm shift reveals lactate's multifaceted roles in tumor biology, far surpassing its former characterization as a metabolic waste product.

The lactate shuttle hypothesis has reshaped understanding, emphasizing lactate's vital role in substrate transport and signaling modulation [2, 3]. Brooks' foundational research illuminated the production and utilization of lactate under aerobic conditions, highlighting its capacity as a signaling molecule [4]. Lactate interacts with cellular receptors, such as G protein‐coupled receptor 81 (GPR81), and is transported via monocarboxylate transporters (MCTs), showcasing its active participation in cellular functions [5].

Metabolic reprogramming in tumor cells, marked by the suppression of mitochondrial oxidative phosphorylation (OXPHOS), represents a hallmark of the Warburg effect [6]. This metabolic shift accommodates the heightened ATP demands of proliferating tumor cells, driving increased glycolysis and consequent lactate accumulation. Elevated lactate levels are a hallmark not only of cancer but also of various inflammatory conditions, underscoring its broad pathophysiological relevance [7].

The discovery of lactylation, a novel post‐translational modification (PTM) driven by lactate, has further elucidated the complex interplay between lactate and tumor biology [8]. Analogous to well‐known modifications like acetylation and methylation, lactylation exerts control over transcriptional regulation, influencing both metabolic and epigenetic landscapes [9]. Pioneering research by Zhao et al. in 2019 revealed lactate's role in histone modification at lysine residues, adding a new dimension to cancer's epigenetic framework [10].

Given these developments, this review aims to consolidate current evidence on the roles of lactate and protein lactylation in tumor progression and immune regulation. We provide a comprehensive overview of lactate metabolism, transport, and signaling, as well as the enzymatic pathways driving lactylation. The regulatory role of histone deacetylases (HDACs) in lactylation is examined in detail. We further explore how lactate and lactylation influence immune homeostasis, epigenetic remodeling, and therapeutic resistance in cancer. Finally, we summarize emerging therapeutic strategies targeting the lactate–lactylation axis, with particular emphasis on their translational potential. This review is structured to guide readers from fundamental biochemical mechanisms to clinical perspectives, offering insight into the metabolic vulnerabilities and epigenetic regulators that define lactate's multifaceted role in cancer biology.

## 2 The Role of Lactate in Tumors

### 2.1 Warbug Effect and Tumor Lactate Metabolism

Tumor‐derived lactate originates primarily from glycolytic reprogramming. This section outlines the mechanisms of lactate production, accumulation, and transport in the tumor microenvironment (TME).

The TME, comprising tumor cells, endothelial cells, cancer‐associated fibroblasts (CAFs), immune cells, and non‐cellular components like the extracellular matrix, is a complex internal milieu. The TME also contains various soluble cytokines secreted by multiple cell types, facilitating intricate interactions within the environment. As early as the 1920s, Otto Warburg, a German scientist, observed that tumor cells, unlike normal cells, rely on elevated glycolysis even in the presence of sufficient oxygen, a phenomenon termed aerobic glycolysis, now a hallmark of tumor metabolism [11].

Lactate, an inevitable by‐product of glycolysis, accumulates in the TME, contributing significantly to its composition. This accumulation stems from both tumor and non‐tumor cells. In aerobic glycolysis, tumor cells preferentially convert glucose to lactate for energy rather than utilizing OXPHOS, leading to substantial lactate production. Lactate levels in tumor cells are estimated to be 40 times higher than in normal cells [12]. Furthermore, glutamine uptake is markedly increased in tumor cells, where it is converted to glutamate by glutaminase. The glutamate enters the mitochondria and participates in the tricarboxylic acid (TCA) cycle as α‐ketoglutarate. The TCA cycle yields malate, which is transported to the cytoplasm and oxidized to pyruvate. Subsequently, lactate dehydrogenase isoform A (LDHA) reduces pyruvate to lactate while regenerating nicotinamide adenine dinucleotide (NAD+). Additionally, non‐tumor cells, such as CAFs, contribute to lactate buildup through glucose uptake and glycolysis.

Lactate transport between cells is primarily mediated by MCTs, members of the solute carrier family SLC16A [13, 14, 15]. Among these, MCT1 (SLC16A1) and MCT4 (SLC16A3) are crucial in cancer metabolism [16, 17]. To prevent excessive intracellular acidification, tumor and non‐tumor cells in hypoxic regions expel glycolysis‐derived lactate into the TME via MCT4. In oxygenated regions, lactate is absorbed by tumor and non‐tumor cells through MCT1 overexpression, providing substrates for the TCA cycle. Concurrently, glucose diffuses into hypoxic areas, sustaining glycolysis in tumor cells. This metabolic symbiosis ensures that tumor cells across different regions meet their energy demands.

Beyond its role as an energy source, lactate functions as a signaling molecule by binding to specific receptors and initiating signal transduction. Recently, GPR81, also known as hydroxycarboxylic acid receptor 1 (HCAR1), was identified as a lactate receptor in adipocytes [18]. GPR81 is predominantly expressed in adipocytes, with significantly lower levels in tissues such as the brain, skeletal muscle, kidney, liver, and macrophages [18]. This receptor contains seven transmembrane domains, to which lactate binds directly, triggering intracellular signaling via the G protein [18]. Upon lactate activation, GPR81 downregulates cyclic AMP, resulting in decreased intracellular cAMP levels [18]. The identification of GPR81 as a lactate sensor highlights the critical role of lactate as a signaling molecule, regulating cellular functions in both normal and pathological contexts.

Together, these mechanisms not only explain the substantial lactate accumulation within the TME but also illustrate how lactate acts as both a fuel and a signaling molecule. Its production, transport, and receptor‐mediated effects provide a metabolic and regulatory foundation for tumor adaptation, laying the groundwork for understanding how lactate influences stromal and immune components of the tumor niche—topics to be explored in the next section.

### 2.2 Lactate and the Tumor Microenvironment

**FIGURE 1:**
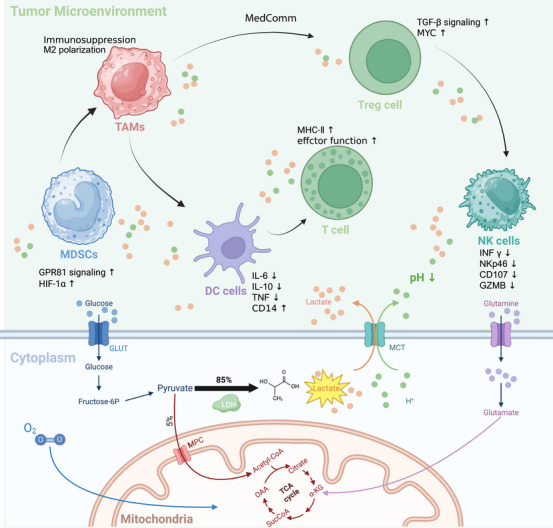
The tumor microenvironment (TME) includes immune and stromal cells, with tumor cells secreting large amounts of lactate, causing an inverse pH gradient and suppressing immune cell function. Lactate, by secreting immunosuppressive molecules and enhancing Treg cell activity, disrupts the immune microenvironment. Additionally, lactate promotes the polarization of M2‐like macrophages, contributing to anti‐inflammatory effects and tumor growth.

Excess lactate establishes an “inverse pH gradient” by raising intracellular pH while lowering extracellular pH, significantly affecting immune cell function and fostering an immunosuppressive environment [19]. This acidified TME promotes cancer cell growth (Figure 1) [20].

#### 2.2.1 T Cells

T cells are particularly sensitive to extracellular lactate, which influences their intracellular signaling, functionality, and overall homeostasis. Elevated lactate levels within the TME impair T‐cell function and immune responses. When the TME's pH drops to between 6.0 and 6.5, CD8+ and CD4+ effector T cells may become anergic, displaying diminished cytotoxic activity and reduced cytokine production [21, 22, 23]. These effector T cells rely on glycolysis for both proliferation and IFN‐γ production [21, 22, 23]. However, lactate inhibits the production of IFN‐γ, TNF‐α, and IL‐2 triggered by T‐cell receptors (TCRs) and diminishes cytotoxic T lymphocyte activity by suppressing p38 MAPK phosphorylation. Moreover, lactate induces T‐cell apoptosis by reducing NAD+ levels [24]. In CD4+ T cells, lactate enhances SIRT1‐mediated deacetylation and degradation of the T‐bet transcription factor, thereby reducing Th1 cell populations [25]. Simultaneously, lactate promotes the differentiation of CD4+ T cells into Th17 cells and impairs T‐cell migration and trafficking [26]. Moreover, recent findings indicate that lactate promotes Th17 differentiation by inducing lysine lactylation at K164 on the transcription factor Ikzf1, which is essential for IL‐17 expression. Pharmacological inhibition of lactate production or protein lactylation using dichloroacetate (DCA) suppresses Th17 development and alleviates inflammation in autoimmune disease models [27].

#### 2.2.2 NK Cells

Natural killer (NK) cells, critical for cancer immunotherapy, exert anti‐tumor effects by secreting pro‐inflammatory cytokines, perforin, and granzymes [28]. However, lactate accumulation within tumor tissues diminishes NK‐cell activity and accelerates tumor immune evasion [29, 30]. Increased lactate levels lower intracellular pH and trigger apoptosis, resulting in decreased NK‐cell cytotoxicity [31, 32]. Furthermore, lactate indirectly affects NK cells by increasing the population of myeloid‐derived suppressor cells (MDSCs).

#### 2.2.3 Dendritic cells (DCs)

Dendritic cells, essential antigen‐presenting cells, are proficient in capturing, processing, and presenting antigens to CD8+ T cells [28]. Lactate disrupts DC differentiation from monocytes, impairs activation, and inhibits antigen processing. It also suppresses cytokine activity in mature DCs, leading to functional impairment. Upon Toll‐like receptor (TLR) stimulation, lactate further hinders DC differentiation by upregulating IL‐10 and reducing IL‐12 production. Research by Gottfried et al. revealed that lactate induces a DC phenotype characterized by reduced IL‐12 secretion and CD1a expression, a hallmark of tumor‐associated dendritic cells (TADCs) observed in melanoma and prostate cancer. Inhibition of lactate production in melanoma models reverses the TADC phenotype. Additionally, lactate accelerates tryptophan catabolism and kynurenine production in plasmacytoid DCs (pDCs) and FoxP3+CD4+ regulatory T cells (Tregs), contributing to immune suppression in the TME [33]. As glycolysis is the primary energy source for pDCs to produce IFN‐γ under TLR stimulation, lactate impairs energy production in pDCs, inhibiting IFN‐γ synthesis [28].

#### 2.2.4 Macrophages

Macrophage phenotypes are influenced by their anatomical location and the surrounding microenvironment [28]. Tumor‐derived lactate directly reprograms cancer‐associated macrophages toward an M2‐like polarization, which supports tumor growth in the TME by secreting immunosuppressive cytokines. This process suppresses the cytotoxic activity of tumor‐infiltrating lymphocytes (TILs) and promotes Treg differentiation [34]. Mechanistically, lactate drives M2 macrophage polarization in breast cancer through multiple pathways, including activation of STAT3 and extracellular signal‐regulated kinase (ERK), upregulation of arginase‐1 (ARG1) and vascular endothelial growth factor (VEGF), and stabilization of HIF‐1α [35]. Lactate also binds to G protein‐coupled receptor 132 (GPR132), further enhancing M2 macrophage polarization.

#### 2.2.5 MDSCs

MDSCs represent a pivotal group of immunosuppressive cells within the TME, playing a critical role in suppressing T‐cell function, proliferation, and TCR signaling while promoting the differentiation of Tregs. The expression profile of glycolytic genes has been shown to correlate with MDSC abundance, both of which are linked to shorter survival times [36]. In triple‐negative breast cancer (TNBC) mouse models, elevated glycolysis and lactate levels promote the development and immunosuppressive functions of MDSCs via stimulation of granulocyte‐macrophage colony‐stimulating factor (GM‐CSF) and granulocyte‐colony stimulating factor (G‐CSF) [36]. Additionally, lactate drives MDSC expansion, which in turn inhibits NK cell activity and limits the function of innate immune effectors. These results highlight lactate's role in fostering a tumor‐immunosuppressive microenvironment by modulating MDSCs, thus facilitating tumor initiation and progression.

These findings underscore lactate's central role in shaping the immunosuppressive landscape of the TME. By modulating various immune cell populations, lactate facilitates tumor immune evasion and supports malignant progression.

### 2.3 Lactate and Tumor

Beyond its impact on immune regulation, lactate directly shapes tumor cell behavior. This section focuses on how lactate contributes to tumor metabolic symbiosis, malignant phenotypes, and immune evasion.

Normal cells metabolize glucose via glycolysis, mitochondrial OXPHOS, and the pentose phosphate pathway. Under normoxic conditions, pyruvate from glycolysis is processed through OXPHOS, converting it into carbon dioxide and water, with glycolysis being downregulated. In contrast, cancer cell metabolism is heterogeneous and varies according to their spatial positioning within the tumor [37]. Cells relying on glycolysis are typically located farther from blood vessels, while those utilizing oxidative metabolism tend to be closer to them. MCTs facilitate the export of lactate and protons, leading to the accumulation of extracellular lactate. Some evidence suggests that excess lactate can be used by other cells as an energy source. Hypoxic cancer cells, situated further from blood vessels, primarily generate energy through glycolysis and release excess lactate into the TME via MCT4. Conversely, cancer cells nearer to blood vessels utilize MCT1 to oxidize lactate and generate ATP under normoxic conditions. This metabolic interplay occurs not only among cancer cells but also in other cell types, such as tumor‐associated endothelial cells and CAFs [21, 38, 39]. Zhou et al. [40] reported that low‐density lipoprotein receptor‐related protein 1 (LRP1) in astrocytes enhances mitochondrial transfer from astrocytes to neurons by reducing lactate production and ARF1 lactylation, thereby mitigating ischemia‐reperfusion injury in a mouse model of ischemic stroke. Similarly, Meng et al. [41] demonstrated that l‐lactate regulates DCBLD1 expression through HIF‐1α activation, promoting the migration, invasion, and proliferation of cervical cancer cells.

Lactate is known to exert an inhibitory effect on effector T cells, aiding tumor cells in immune evasion. In contrast, lactate serves as both an energy source and a modulator of intracellular pH in Tregs, influencing their suppressive function and proliferation [21]. In tumors with high glycolytic activity, particularly those exhibiting elevated MYC expression, Tregs extensively uptake lactate, which enhances PD‐1 expression [42]. Notably, inhibiting lactate uptake by Tregs has been shown to reduce their immunosuppressive capabilities in tumors. Consequently, lowering lactate levels in cancer cells may synergize with immunotherapies, such as anti‐PD‐1 treatments [21].

These insights highlight lactate's dual role as both a metabolic substrate and immunomodulator within tumors, offering rationale for combining lactate‐lowering strategies with immunotherapy.

## 3 The Role of Protein Lactylation in Cancer

### 3.1 Discovery and Mechanism of Protein Lactylation

The identification of protein lactylation has significantly expanded the functional landscape of lactate beyond metabolism, positioning it as an epigenetic regulator. This section outlines the discovery, distribution, and regulatory mechanisms of lysine lactylation on histone and non‐histone proteins.

#### 3.1.1 Discovery

Lactate, a byproduct of glycolysis, plays a pivotal role in energy regulation, wound healing, and the progression of tumor growth and metastasis [12]. Advances in mass spectrometry have significantly expanded the understanding of protein PTMs, uncovering new types of modifications and modification sites. In 2019, Zhao Yingming's team identified a novel histone modification known as histone lysine lactylation [10]. Zhang et al. [10] revealed that lysine lactylation is highly enriched in gene promoter regions and positively correlates with mRNA levels. They also compared lactylation to acetylation, finding that many genes lacking acetylation still exhibited lactylation, suggesting distinct regulatory roles for these modifications.

While lactylation was initially discovered on histones, subsequent research identified its presence on non‐histone proteins as well. Wan et al. [43] observed the formation of cyclic lactylammonium ions during tandem mass spectrometry, which can be used to detect protein lactylation. Their large‐scale analysis of affinity‐enriched lactylated proteomes and non‐lactylated protein spectral libraries established the sensitivity and specificity of this ion for identifying lactylation sites. Using this ion‐based approach, they uncovered novel lactylation sites beyond histones, including those present in non‐enriched human proteome databases. Notably, lactylation was found to be prevalent in glycolytic enzymes, with ALDOA exhibiting conserved lactylation sites. In addition, lactylation was widely observed on DHRS7 in the human tissue proteome draft, highlighting its functional significance. For example, site‐specific lactylation on ALDOA resulted in enzyme inhibition, suggesting a lactylation‐dependent feedback mechanism in glycolysis. Gao et al. [44] conducted the first proteomic analysis of lactylation in the destructive fungal pathogen *Botrytis cinerea*, identifying 273 Kla sites across 166 proteins through LC‐MS/MS, including four distinct modification motifs. The study also evaluated non‐histone protein lactylation in *B. cinerea*. Furthermore, Qiao et al. [45] discovered a novel lactylation modification on the protein Ezrin, with lactylation primarily occurring at the K263 site. Their findings suggested that histone H3K18 lactylation (H3K18la) plays a pivotal role in sepsis‐associated acute kidney injury (SA‐AKI).

#### 3.1.2 Visualization of Lactylation Based on Bibliometrics

**FIGURE 2:**
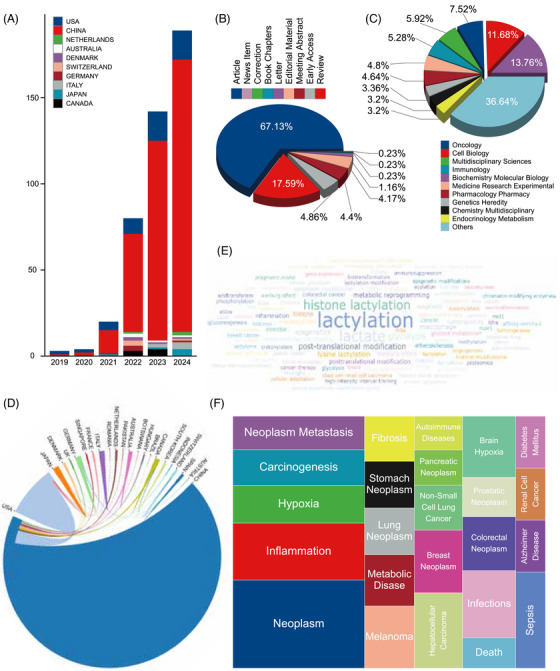
Visualization of lactylation based on bibliometics. (A) Annual changes in the number of articles by country; (B) lactylation research papers by document type percentage; (C) distribution of different research fields of papers for lactylation; (D) collaborative relationships between different countries; (E) keyword cloud; and (F) distribution map of related diseases.

The burgeoning field of lactylation research, initiated by the discovery of histone lactylation in 2019, has experienced a substantial surge in publications. A thorough analysis of the Web of Science Core Collection (WoSCC) database from 2019 to August 8, 2024, reveals 410 publications, underscoring the rapidly growing interest in this area. The statistical analysis demonstrates the exponential rise in lactylation research since its inception (Figure 2A). Notably, Chinese researchers have made significant contributions, as evidenced by the collaborative networks in Figure 2D.

To capture the diverse aspects of lactylation research, a polynomial model categorized publications into nine distinct study types. Original research articles constituted 67.13% of the total, making up the majority, followed by review articles at 17.59% (Figure 2B). The research landscape is primarily focused on biochemistry and molecular biology, with significant contributions from cell biology, oncology, multidisciplinary sciences, and immunology (Figure 2C).

Keyword analysis from Figure 2E,F highlights a wide range of research interests, particularly in metabolism, immunity, and protein PTMs. Cancer research has emerged as a prominent focus, alongside studies on inflammation, hypoxia, infection, and Alzheimer's disease.

These bibliometric insights offer a foundation for synthesizing the latest advancements in lactylation, aiming to provide a comprehensive overview of its role in tumor biology and establish directions for future investigations.

#### 3.1.3 Lysine Lactylation

Recent studies have revealed that cellular lactate levels are tightly correlated with the global abundance and site specificity of lysine lactylation. Under high glycolytic activity, elevated intracellular lactate can be enzymatically converted to lactyl‐CoA, a critical donor substrate for histone and non‐histone lactylation. Key histone marks such as H3K18la [45, 46, 47, 48, 49, 50, 51] and H4K12la [52, 53, 54] are deposited in response to lactyl‐CoA availability and regulate transcriptional programs. Moreover, non‐histone proteins like HMGB1 [55] and p53 [56] are also lactylated in a lactate‐dependent manner. Notably, lactate‐sensing enzymes such as AARS1 can bypass the need for lactyl‐CoA by directly forming a lactyl‐AMP intermediate and catalyzing site‐specific lactylation [56, 57, 58]. These findings suggest that lactate acts not only as a metabolic by‐product but also as a dynamic regulator of the posttranslational modification landscape.

Advances in research techniques, such as liquid chromatography‐tandem mass spectrometry, have led to the identification of numerous lysine lactylation (Kla) sites across both eukaryotic and prokaryotic organisms. For instance, 26 histone lactylation sites were identified in HeLa cells, while 16 sites were discovered in mouse bone marrow‐derived macrophages [10]. In liver cancer tissues, 9275 Kla sites were identified, 9256 of which were located on non‐histone proteins. Additionally, 2375 Kla sites were identified across 1014 proteins in AGS cells (a gastric cancer cell line), while lung tissue revealed 724 Kla sites in 141 proteins [59]. In prokaryotes, a quantitative proteomic analysis of *Escherichia coli* uncovered 446 endogenous Kla sites targeted by CobB and 79 candidate sites targeted by YiaC [60]. In the fungal pathogen *B. cinerea*, 273 lactylation sites were identified across 166 proteins, with distribution in the nucleus (36%), mitochondria (27%), and cytoplasm (25%) [61]. Meng et al. [62] presented the first global lactylome of rice, identifying 638 lysine lactylation sites across 342 proteins in rice grains. An et al. [63] presented comprehensive lactylome of *Frankliniella occidentalis*, identifying 1458 lysine lactylation sites across 469 proteins, with functional enrichment in translation, protein folding, and energy metabolism. Yin et al. [54] performed the first global lactylome profiling in *Toxoplasma gondii*, identifying 1964 Kla sites on 955 proteins including histones H3K14la and H4K12la. The discovery of these sites highlights the regulatory potential of lactylation modifications across various biological pathways, underscoring their importance for future research.

In addition to mapping lysine lactylation sites across species, recent advances have distinguished the stereochemical variants of this modification. Zhang et al. [64] employed chiral derivatization and isomer‐specific antibodies to demonstrate that lysine l‐lactylation (Kl‐la), rather than d‐lactylation (Kd‐la) or N‐ε‐carboxyethylation (Kce), represents the predominant isomer on histones and is dynamically regulated by glycolytic flux. These findings not only confirm Kl‐la as a key metabolically responsive epigenetic mark but also underscore the importance of resolving structural isomers in future lactylation research.

#### 3.1.4 Regulation of Lysine Lactylation

**FIGURE 3:**
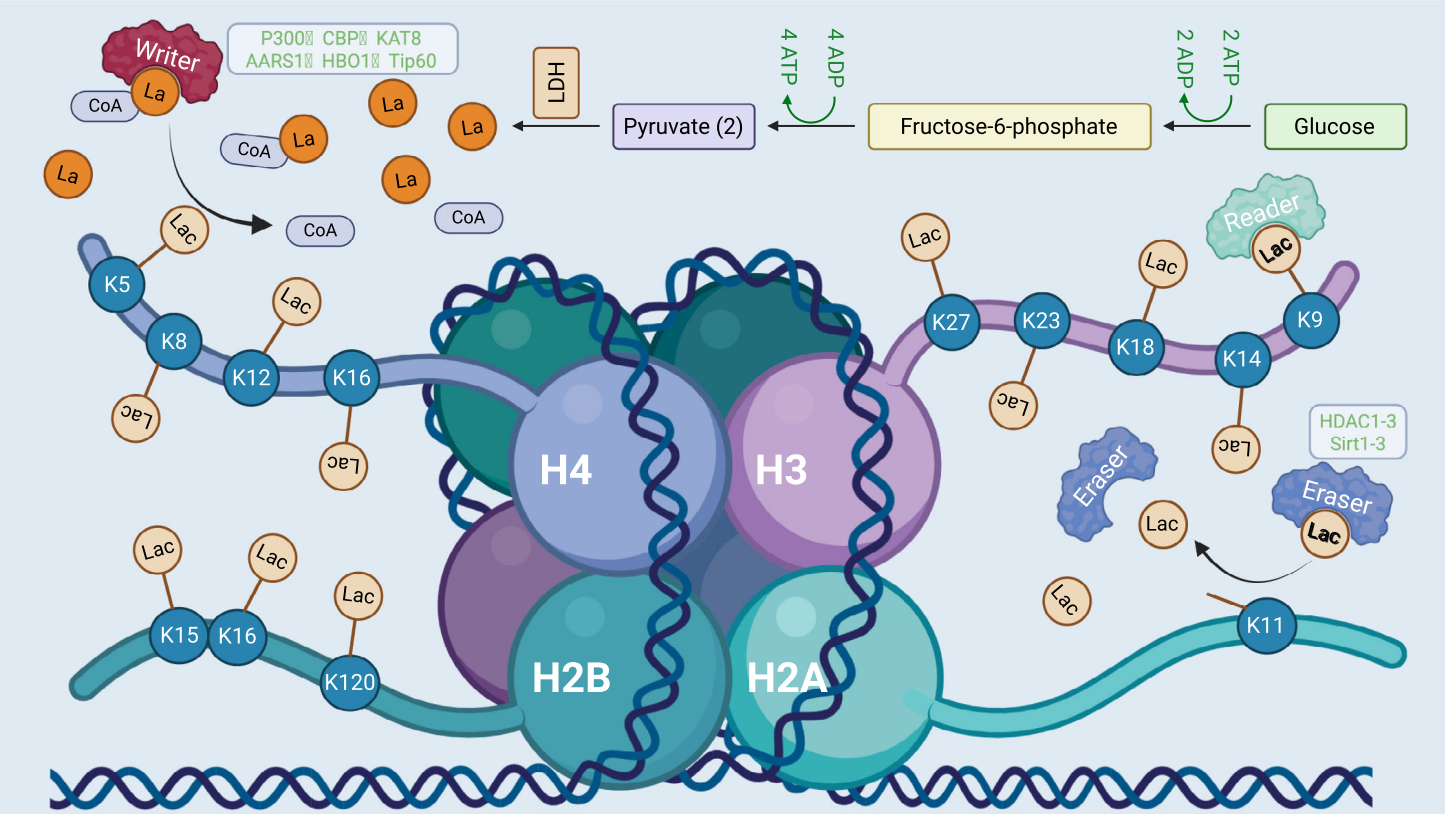
Lactylation at the histone amino terminus. The figure depicts DNA coiled around a histone octamer, forming nucleosomes composed of two each of H2A, H2B, H3, and H4 histones. The histone tails, which protrude from the nucleosomal core, are susceptible to diverse post‐translational modifications (PTMs), including lactylation (Lac). These modifications not only modulate the chromatin structure's overall compaction but also influence gene expression. Lactylation is dynamically regulated by “writers” (e.g., p300, CBP, AARS1) that catalyze the addition of lactyl groups, “erasers” (e.g., HDAC1‐3 and SIRT1‐3) that remove them, and “readers” that recognize lactylated lysines to mediate transcriptional and chromatin responses. All three classes of regulators are depicted in the figure.

Zhang et al. [10] proposed an intriguing function for the Warburg effect beyond energy production, suggesting that lactate serves as a precursor for generating lactyl‐CoA, which facilitates lysine lactylation on histones, thereby promoting gene expression. This histone lactylation is a reversible process regulated by specific enzymes, rather than occurring spontaneously. This reversible modification is orchestrated by three categories of regulatory proteins: “writers” that catalyze the addition of lactyl groups to lysine residues, “erasers” that remove these modifications, and “readers” that specifically bind to lactylated lysine residues and mediate transcriptional regulation or chromatin remodeling. An in vitro chromatin reconstitution experiment demonstrated that histone lactylation can directly drive gene transcription through mechanisms involving p53 and p300 [10]. Lactate triggers histone lactylation via the “writer” p300, activating gene transcription [10]. Both class I HDACs (HDAC1‐3) and class III HDACs (SIRT1‐3) possess de‐lactylation capabilities, with class I HDACs, particularly HDAC1 and HDAC3, showing the strongest de‐lactylation activity [65, 66]. Among these, NAD+‐dependent deacetylase SIRT3 plays a critical role in inhibiting hepatocellular carcinoma (HCC) progression by de‐lactylating non‐histone proteins (Figure 3) [67]. Fan et al. [68] identified SIRT3 as the “eraser” of H4K16la. Chen et al. [69] revealed that lactate‐induced lactylation of NBS1 enhances DNA repair via homologous recombination (HR), with TIP60 identified as the “writer” and HDAC3 as the “eraser” of NBS1 K388 lactylation. Sun et al. [70] demonstrated that HDAC6 acts as a primary lactyltransferase catalyzing lactate‐dependent α‐tubulin K40 lactylation. In prokaryotes, YdiF catalyzes the formation of lactate‐CoA, which YiaC uses to attach lactyl groups to specific proteins, while CobB subsequently removes these lactyl groups; CobB demonstrates de‐lactylation activity both in vitro and in vivo [60]. Zong et al. [56] identified AARS1 as a lactate sensor that regulates global lysine lactylation in tumor cells, showing that lactylation of p53 at K120 and K139 by AARS1 impairs the liquid–liquid phase separation (LLPS) and DNA binding capacity of p53. Li et al. [58] identified AARS1 and AARS2 as intracellular l‐lactate sensors and global lysine lactyltransferases, and showed that AARS2‐mediated cGAS lactylation suppresses DNA sensing and innate immunity. Furthermore, both AARS1 and SIRT3 are recognized as key regulators of OXPHOS, the primary ATP production pathway dependent on oxygen [71]. Mao et al. [71] demonstrated that lactylation of mitochondrial proteins integrates hypoxic and lactate signals to modulate OXPHOS. Hu et al. [72] further revealed that Brg1 binds to H3K18la, indicating a novel role for Brg1 as a “reader” of histone lactylation. Additionally, Niu et al. [73] identified HBO1 as a lysine lactyltransferase that regulates transcription, while Yan et al. [74] discovered that ATAT1 functions as the writer of NAT10 lactylation.

In summary, protein lactylation is a newly recognized PTM that bridges cellular metabolism and gene regulation. Its widespread presence across histone and non‐histone proteins, together with its dynamic enzymatic regulation, lays a mechanistic foundation for its emerging roles in modulating the TME.

### 3.2 Protein Lactylation and the Tumor Microenvironment

Lactate accumulation in the TME is known to orchestrate immune suppression through diverse mechanisms. Recent discoveries have revealed that, beyond metabolic effects, protein lactylation acts as a crucial epigenetic mediator driving immunosuppressive cell states. This section focuses on the emerging roles of histone and non‐histone lactylation in reshaping immune cell behavior within the TME.

#### 3.2.1 Macrophage Polarization

In tumors, macrophages can adopt either a pro‐inflammatory (M1) or anti‐inflammatory (M2) phenotype. Zhang et al. [10] demonstrated that bacterial components, such as lipopolysaccharide (LPS), can induce M1 macrophage polarization, characterized by the activation of inflammatory genes, increased glycolysis, and elevated lactate production. Interestingly, they found that M2 macrophages exhibited significantly higher levels of histone lactylation, alongside increased glycolysis and intracellular lactate. While inflammation‐related genes were rapidly upregulated following LPS exposure, this response was not directly correlated with lactate levels or lysine lactylation. Instead, lysine lactylation occurred more gradually and was associated with the activation of housekeeping genes involved in maintaining cellular homeostasis. Zhang et al. [10] also reported that stimulating bone marrow‐derived macrophages with LPS and interferon‐γ to induce M1 polarization resulted in a time‐dependent increase in histone lactylation. Adding exogenous lactate to M1 macrophages further enriched lysine lactylation at the Arg1 promoter, boosting gene expression. Conversely, the absence of LDHA in M1 macrophages reduced both histone lactylation and Arg1 expression. Notably, tumor‐associated macrophages (TAMs) exhibited higher levels of histone lysine lactylation compared to those in other tissues.

Emerging evidence suggests that metabolic reprogramming is essential for the transition between macrophage phenotypes. Pyruvate kinase M2 (PKM2) plays a key role in this metabolic adaptation in pro‐inflammatory macrophages. PTMs are essential for regulating PKM2 activity. Wang et al. [75] discovered that lactate modulates the Warburg effect by lactylating PKM2 at the K62 site, which inhibits its conversion from a tetramer to a dimer, thereby enhancing its pyruvate kinase activity and reducing its nuclear localization. Furthermore, lactate‐induced lactylation of PKM2 was shown to reprogram pro‐inflammatory macrophages into reparative macrophages, highlighting the role of lactylation in macrophage phenotype regulation.

#### 3.2.2 MDSCs

Elevated lactate levels promote the development and immunosuppressive functions of MDSCs, enhancing the production of G‐CSF and GM‐CSF [36]. Lactate also accelerates MDSC proliferation, contributing to the creation of an immunosuppressive microenvironment that supports tumor initiation and progression by modulating MDSCs.

#### 3.2.3 Treg Cells

Gu et al. [76] examined the effects of lactate on Tregs and found that lactate increases Forkhead box protein P3 (FOXP3) expression, improving the stability and function of Tregs. Conversely, reducing lactate levels can suppress Tregs and activate anti‐tumor immunity. Mechanistically, lactate facilitates Treg cell generation by lactylating MOESIN at Lys72, which strengthens the interaction between MOESIN and transforming growth factor‐beta receptor I (TGF‐βRI), thereby regulating downstream SMAD3 signaling.

Other studies have also shown that the TME enhances Treg recruitment and differentiation by increasing FOXP3 and MCT1 expression [28]. Similarly, elevated lactate levels secreted by glycolytic tumors support immune evasion by maintaining Treg immunosuppressive function and weakening effector T cells [77].

#### 3.2.4 DCs

In the TME, lactate accumulation has been shown to suppress the development of pro‐inflammatory DCs and favor the formation of tolerogenic dendritic cells (tolDCs). This alters the antigen‐presenting capabilities of DCs, inhibiting T‐cell activation and proliferation, which suppresses the immune response against tumors. Studies indicate that lactate exposure increases interleukin‐10 (IL‐10) secretion by DCs while reducing the secretion of pro‐inflammatory cytokines such as interleukin‐12 (IL‐12), tumor necrosis factor (TNF), and interleukin‐23 (IL‐23). This shift in cytokine profile fosters immune evasion by tumor cells. Moreover, lactate impairs DC maturation and function by downregulating surface markers like CD1a and upregulating CD14 and CD86. These changes in DCs contribute to the tumor's ability to escape immune surveillance.

Collectively, these findings reveal that protein lactylation actively participates in constructing an immunosuppressive TME by regulating multiple immune cell subsets. From promoting M2 macrophage polarization and expanding MDSCs to enhancing Treg stability and impairing DC activation, lactylation functions as a metabolic‐epigenetic switch that links elevated lactate levels to immune escape. These insights highlight lactylation as a promising target for reprogramming the tumor immune landscape in cancer therapy.

### 3.3 Protein Lactylation in Non‐Oncologic Diseases

Recent studies have revealed that protein lactylation also participates in a wide array of non‐cancerous pathological processes.

An et al. [78] revealed that lactate‐induced non‐histone lactylation of Fis1 promotes mitochondrial dysfunction and exacerbates sepsis‐induced acute kidney injury. Yang et al. [55] demonstrated that lactate induces HMGB1 lactylation and acetylation via p300/CBP and GPR81 signaling, promoting its exosomal release and endothelial dysfunction in sepsis. Wang et al. [53] demonstrated that PFKFB3 promotes kidney fibrosis by enhancing glycolysis‐driven lactate accumulation and histone H4K12 lactylation, which epigenetically activates NF‐κB pathway genes (Ikbkb, Rela, and Relb) and aggravates renal inflammation and fibrogenesis. Dong et al. [49] demonstrated that ASF1A‐dependent P300‐mediated histone H3K18 lactylation promotes atherosclerosis by activating SNAI1 transcription and driving EndMT in endothelial cells. Li et al. [52] demonstrated that TRAP1 promotes vascular smooth muscle cell senescence and atherosclerosis by enhancing glycolysis and lactate accumulation, which suppresses HDAC3 expression and increases H4K12la at SASP gene promoters, thereby activating senescence‐related transcription and plaque progression. Caielli et al. [79] identified that lactate‐induced lactylation of proteasome subunits disrupts mitochondrial clearance, promoting IFN‐I responses in systemic lupus erythematosus. Also, Zhang et al. [80] demonstrated that mtDNA‐induced lactate promotes cGAS lactylation, which blocks its ubiquitination by MARCHF5 and enhances IFN‐I responses in SLE. Fan et al. [81] demonstrated that lactate induces Snail1 lactylation via MCT‐dependent transport, thereby activating TGF‐β/Smad2 signaling and promoting endothelial‐to‐mesenchymal transition (EndoMT) and cardiac fibrosis following myocardial infarction. Yao et al. [82] identified 1003 lysine lactylation sites in the cerebral endothelium of ischemia–reperfusion rats and showed that lactylation dysregulation affects mitochondrial apoptosis pathways—especially via Slc25a4, Slc25a5, and VDAC1—involved in Ca^2^⁺ signaling, contributing to neuronal injury. Wang et al. [51] demonstrated that lactate‐induced H3K18 lactylation in alveolar epithelial cells promotes idiopathic pulmonary fibrosis progression by upregulating YTHDF1, enhancing m6A modification of NREP, and activating TGF‐β1‐mediated fibroblast‐to‐myofibroblast transition. Rho et al. [83] demonstrated that HK2‐driven lactate production promotes H3K18 lactylation in hepatic stellate cells, which activates fibrosis‐related gene expression and drives liver fibrosis. Lin et al. [84] demonstrated that hypoxia‐induced glycolysis elevates lactate production and histone H3K18 lactylation in the sclera, which activates Notch1 transcription and drives fibroblast‐to‐myofibroblast transdifferentiation, ultimately promoting myopia development. Ma et al. [85] demonstrated that NR4A3 promotes vascular calcification by enhancing glycolysis and lactate production, which induces p300‐mediated histone H3K18 lactylation and upregulates Phospho1 expression, driving osteogenic reprogramming in vascular smooth muscle cells. Wei et al. [47] demonstrated that senescent microglia exhibit elevated glycolysis‐derived lactate levels and H3K18 lactylation, which epigenetically activate the NF‐κB pathway by enhancing binding to Rela and NFκB1 promoters, thereby upregulating IL‐6 and IL‐8 and accelerating brain aging and Alzheimer's disease progression.

In contrast to its pathogenic roles, accumulating evidence also suggests that protein lactylation can exert protective or reparative effects under specific physiological or pathological conditions. Wang et al. [86] demonstrated that histone H3K18 lactylation in monocyte–macrophages promotes early activation of reparative genes such as Lrg1, Vegf‐a, and IL‐10 following myocardial infarction, thereby enhancing anti‐inflammatory and proangiogenic responses, improving cardiac repair and function. Wang et al. [87] demonstrated that exercise promotes lysine lactylation of Mecp2 at K271 in endothelial cells, which inhibits Ereg transcription and suppresses Egfr/MAPK pathway activation, thereby reducing vascular inflammation and attenuating the progression of atherosclerosis.

Collectively, these studies illustrate the duality of protein lactylation in non‐oncologic diseases, acting as either a pathogenic driver or a protective modulator depending on cellular context and disease stage. This bidirectional regulatory capacity suggests that targeting lactylation may hold therapeutic promise not only in cancer but also in chronic inflammatory, metabolic, and degenerative disorders.

### 3.4 Protein Lactylation and Cancer

As lactate accumulates in the TME due to aberrant glycolysis, it not only fuels metabolic crosstalk but also drives PTMs that shape tumor fate. Among these, protein lactylation has emerged as a novel epigenetic mechanism linking cellular metabolism to oncogenic regulation. Recent advances have revealed its widespread involvement in tumorigenesis, progression, and metabolic rewiring across cancer types, offering new perspectives on how metabolic byproducts may encode epigenetic instructions for malignancy.

#### 3.4.1 Tumorigenesis

Yu et al. demonstrated that elevated histone lactylation correlates with poor prognosis in patients with ocular melanoma, and inhibiting this modification can suppress tumor progression. The study further identified YTHDF2 as a novel oncogene, whose transcription is directly upregulated by H3K18la enrichment at its promoter. Once induced, YTHDF2 binds m6A‐modified transcripts of tumor suppressor genes such as PER1 and TP53, enhancing their decay. This process reduces tumor suppressive signaling and promotes immune evasion and cell survival, thereby contributing to malignant transformation at the post‐transcriptional level [76]. Zong et al. [56] additionally found that AARS1 functions as both a lactate sensor and a lactyltransferase in tumor cells. It binds lactate to form a lactyl–AMP intermediate, which is then used to directly lactylate lysine residues on substrates such as p53. Lactylation of p53 at specific lysine sites (e.g., K382) disrupts its tetramer formation and reduces its transcriptional activity, weakening its ability to activate target genes such as CDKN1A and BAX. This modification thereby impairs p53‐mediated cell cycle arrest and apoptosis, removing a key barrier to tumorigenesis. Moreover, AARS1‐mediated lactylation shows wide substrate specificity, suggesting its role in linking tumor metabolism with proteomic changes, thus contributing to tumor development. Yang et al. [88] identified 2375 lysine lactylation sites across 1014 proteins in gastric cancer cells and demonstrated that lactylated proteins are enriched in spliceosome pathways, with elevated Kla levels predicting poor prognosis, suggesting a regulatory role for lactate in RNA splicing and tumor progression. In addition, Gu et al. [89] demonstrated that H3K18 lactylation upregulates ALKBH3 expression, which in turn mediates SP100A demethylation, impairs the formation of promyelocytic leukemia (PML) nuclear bodies, and thereby facilitates tumor progression. Liao et al. demonstrated that CENPA [90] undergoes lactylation at K124, which enhances its transcriptional activity and promotes HCC progression through forming a co‐transcriptional complex with YY1 to upregulate CCND1 and NRP2 expression. Similarly, Chen et al. [91] found that lactate‐induced H3K18la promotes the transcription of NSUN2, an RNA methyltransferase that enhances ENO1 expression through m5C modification. NSUN2 is also lactylated at K356, which strengthens its RNA‐binding ability and reinforces glycolysis and lactate production, forming a positive feedback loop that drives colorectal cancer progression. Qiao et al. [92] demonstrated that hypoxia‐induced lactylation of SHMT2 enhances its protein stability, promotes MTHFD1L expression, and facilitates glycolysis and stemness in esophageal cancer cells, revealing an SHMT2 lactylation–MTHFD1L axis driving malignant progression under hypoxic conditions.

#### 3.4.2 Tumor Progression

**FIGURE 4:**
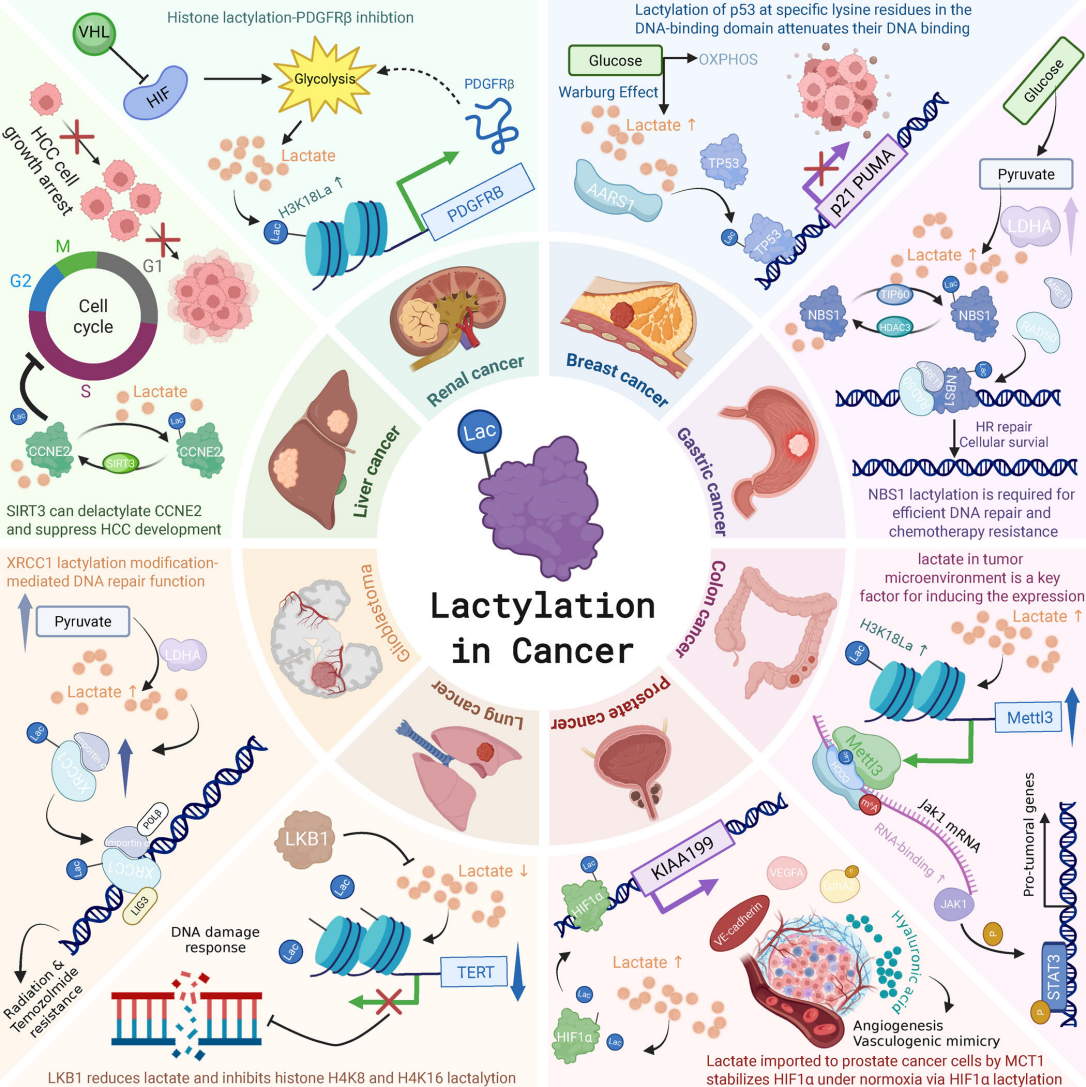
The mechanism by which protein lactylation modification promotes tumor progression in several types of cancer.

Histone lactylation has been implicated in promoting tumor progression (Table 1; Figure 4). Platelet‐derived growth factor receptor β (PDGFRβ), a transmembrane receptor tyrosine kinase, has been identified as a key target gene for histone lactylation in clear cell renal cell carcinoma (ccRCC) [93]. Upon ligand binding, PDGFRβ undergoes phosphorylation and internalization, activating multiple docking sites for downstream signaling [94]. Elevated PDGFRβ levels have been observed in various cancers, including gastrointestinal, lung, breast, hepatocellular, and pancreatic cancers [95]. Activation of PDGFRβ signaling drives tumor proliferation, metastasis, and angiogenesis through the PI3K/AKT and Ras/MAPK pathways [96]. Yang et al. [97] discovered that histone lactylation, triggered by inactive von Hippel–Lindau (VHL), promotes ccRCC progression via PDGFRβ activation. In turn, PDGFRβ signaling enhances glycolysis, lactate production, and histone lactylation, establishing a feedback loop that accelerates ccRCC progression.

**TABLE 1:** Lactate promotes tumor progression.

Cancer type	Study	Cell lines	Relevanta protein lactylation
Ocular melanoma	Yu et al. [98]	OCM1, CRMM1	H3K18la
Hepatocellular cancer	Pan et al. [99]	LCSCs	H3K91a, H3K56la
	Yang et al. [59]	HepG2, Hep3B	AK2‐K28la
	Jin et al. [67]	Huh7	K347la, K348la
Colon cancer	Xiong et al. [100]	TIMs	H3K18la
	Chen et al. [91]	SW480, HT29	H3K18la
	Li et al. [101]	THP‐1, RAW264.7, BM‐M4s, HCT116, SW480, MC38	H3K18la
	Li et al. [48]	HCT116, SW620	H3K18la
	Zhou et al. [102]	HCT116, HT29	H3K18la
	Wang et al. [103]	HCT116	H4K8la
	Miao et al. [104]	SW620, RKO	β‐Catenin lactylation
	Xie et al. [105]	HCT116, SW480	eEF1A2‐K408la
	Zhao et al. [106]	LoVo, DLD‐1	
Non‐small cell lung cancer	Jiang et al. [107]	A549, H1299	H4K8la
Gastric cancer	Ju et al. [57]	HGC27	H3K18la, YAPK90la, TEAD1K108la
	Sun et al. [108]	HGC27, AGS	METTL16‐K229la
Pancreatic ductal adenocarcinoma	Li et al. [109]	MIA PaCa‐2, AsPC‐1, PANC‐1, PL45	H3K18la
Glioblastoma	Li et al. [110]	N33 cells, GSCs, organoids	XRCC1‐K247la
	Yue et al. [111]	TBD0220/TR, U87/TR	H3K9la
	Zhu et al. [112]	U251, U87, GSC3565, GSC387, CT2A	H3K18la, H3K14la
Intrahepatic cholangiocarcinoma	Yang et al. [113]	RBE, HCCC9810, HuCCT1, QBC939	NCL‐K477la
Bladder cancer	Li et al. [114]	T24/T24R, SW780/SW780R	H3K18la
	Xie et al. [115]	T24, TCCSUP	H3K18la
Neuroendocrine prostate cancer	Wang et al. [116]	LNCaP, TRAMP‐C1	H3K18la
Cervical cancer	Meng et al. [41]	HeLa, C33A, SiHa	DCBLD1‐K172la
	Meng et al. [117]	C33A, SiHa, PHKs	G6PD‐K45la
Oral squamous cell cancer	Jing et al. [118]	CAL27, LEUK1	DHX9‐K146la
Breast cancer	Zhang et al. [10]	MCF‐7	H3K18la, H4K12la
	Hou et al. [119]	MDA‐MB‐231, MCF‐7, MDA‐MB‐468, T47D	H3K18la
	Pandkar et al. [120]	MCF7, HCC1806	H3K18la

In prostate cancer, Luo et al. [121] found that the KIAA1199 gene promotes tumor development by stimulating angiogenesis through hypoxia and glycolysis. Elevated KIAA1199 levels correlate with increased HIF1α and enhanced angiogenesis. Lactate, transported into prostate cancer cells via MCT1, further stimulates KIAA1199‐mediated angiogenesis and hyaluronic acid degradation.

In colorectal cancer, Chu et al. [122] revealed that ALDOB‐driven lactate production enhances CEACAM6 protein lactylation, stabilizing CEACAM6 and promoting tumor cell proliferation and 5‐FU resistance. Sun et al. [123] demonstrated that SMC4 knockdown induces a diapause‐like state in colorectal cancer cells characterized by low proliferation and chemotherapy resistance, driven by glycolysis‐dependent lactate production and H4K12 histone lactylation, which upregulates ABC transporter genes and promotes drug efflux.

Meng et al. [41] reported that l‐lactate regulates the expression of discoidin, CUB, and LCCL domain containing 1 (DCBLD1) via HIF‐1α activation and that l‐lactate inhibits DCBLD1 degradation by directly enhancing DCBLD1 lactylation [41].

Liu et al. [124] identified GTPSCS as a nuclear lactyl‐CoA synthetase that interacts with p300 to form a functional lactyltransferase complex, catalyzing histone H3K18 lactylation and upregulating GDF15 expression, thereby promoting glioma proliferation and radioresistance. Zhu et al. demonstrated that ACSS2 synthesizes lactyl‐CoA from lactate and forms a complex with KAT2A to act as a histone lactyltransferase, promoting H3K18/K14 lactylation, gene expression, glio [112] blastoma growth, and immune evasion.

Similarly, Huang et al. [125] observed that the lactylation of Yin‐Yang 1 (YY1) exacerbates microglial dysfunction in autoimmune uveitis by increasing inflammatory cytokine secretion and promoting cell migration and proliferation.

However, not all lactylation events promote disease progression. Meng et al. [117] recently reported that lactylation of glucose‐6‐phosphate dehydrogenase (G6PD) at lysine 45 suppressed cervical cancer cell proliferation by reducing pentose phosphate pathway activity. In contrast, the HPV16 E6 oncoprotein was shown to promote tumor growth by inhibiting this specific lactylation modification. These findings highlight the context‐dependent roles of protein lactylation in cancer biology.

#### 3.4.3 Tumor Metabolism

Li et al. [110] demonstrated that the interaction between ALDH1A3 and PKM2 enhances PKM2 tetramerization, leading to increased lactate production and XRCC1 lactylation at lysine 247 (K247) in glioblastoma stem cells (GSCs). Elevated lactylation of XRCC1 improves its binding to importin α, facilitating nuclear translocation and enhancing DNA repair.

Furthermore, Merkuri et al. [126] reported that glycolysis‐regulated histone lactylation links the metabolic state of embryonic cells to chromatin organization and gene regulatory network (GRN) activation. The effects of protein lactylation on various tumors are summarized in Figure 4.

Collectively, protein lactylation serves as a pivotal integrator of oncogenic metabolism and epigenetic regulation. It modulates diverse cancer hallmarks by altering chromatin states, stabilizing oncogenic proteins, and fine‐tuning metabolic gene expression. While most current evidence supports a pro‐tumorigenic role, context‐dependent tumor‐suppressive functions have also emerged. These findings underscore lactylation as a promising yet complex therapeutic target. Further investigations are warranted to delineate its dynamic regulation, substrate specificity, and potential for clinical intervention.

## 4 Potential Therapeutic Strategies

### 4.1 Targeting Lactate Metabolism and Lactate Transport

Targeting lactate metabolism offers new therapeutic opportunities in cancer treatment. As lactate drives tumor progression and immune suppression, interventions aimed at its production, transport, and signaling have gained attention. This section outlines key strategies and emerging agents that modulate the lactate–lactylation axis.

#### 4.1.1 Inhibiting LDH

Inhibiting lactate production by targeting lactate dehydrogenase (LDH) or other glycolytic enzymes offers a potential strategy to enhance cancer therapies. Elevated LDH levels in patients with melanoma have been associated with a reduced response to anti‐PD‐1 immunotherapy [127]. Research in mouse melanoma models suggests that blocking LDHA can boost antitumor immune responses by increasing the production of IFN‐γ and granzyme B in CD8^+^ T cells and NK cells, thus improving the efficacy of PD‐1 inhibitors [128]. However, as inhibitors of glycolytic enzymes are still in preclinical stages, their clinical impact remains uncertain. Another study showed that inhibiting LDHA with GSK2837808A in patient‐derived and B16 melanoma models enhances T‐cell function both in vitro and in vivo, while also improving the effectiveness of adoptive cell therapy [129]. Chen et al. [69] revealed that elevated NBS1 K388 lactylation is linked to poor outcomes in neoadjuvant chemotherapy. Reducing lactate levels through genetic depletion of LDHA or treatment with stiripentol, a clinically approved LDHA inhibitor for epilepsy, decreases NBS1 K388 lactylation, reduces DNA repair efficiency, and helps overcome chemotherapy resistance. Stiripentol, identified as a lactylation inhibitor, also increases the sensitivity of glioblastoma (GBM) cells to temozolomide (TMZ) in both in vitro and in vivo models, suggesting a combined therapeutic approach for GBM treatment [111].

#### 4.1.2 Inhibiting MCTs

Lactate levels in the TME can also be reduced by inhibiting its transport. MCTs facilitate lactate transport, with MCT1 having the highest affinity for lactate and functioning as a bidirectional transporter, while MCT4 is more highly expressed in glycolysis‐intensive tissues, including tumors [130]. Various MCT1 and MCT4 inhibitors, such as AZD3965, 7ACC2, AR‐C155858, and syrosingopine, have been developed in preclinical studies, though only AZD3965 is currently in clinical trials (NCT01791595) [28].

In HCC, elevated MCT4 levels correlate with poor prognosis, and in mouse HCC models, excessive MCT4 expression suppresses CD8+ T cell activity [131]. MCT4 inhibitors counteract TME acidification and enhance chemokine expression, such as CXCL9 and CXCL10, through the ROS/NF‐κB signaling pathway.

#### 4.1.3 Blocking the Lactate Cycle

Addressing the acidic nature of the TME through pH modulation has also been explored. Research on B16 melanoma demonstrated that bicarbonate administration reduces tumor growth, increases CD8+ T cell infiltration, and enhances the activation of NK and B cells. This approach improves the efficacy of anti‐CTLA4, anti‐PD‐1 therapies, and adoptive cell therapy [100, 132].

In tumors, metabolites in the TME significantly impair immune cell function, especially those targeting tumor cells. As a result, metabolic therapies that adjust glucose metabolism to reshape the TME could become valuable adjuncts to immune checkpoint inhibitors (ICIs). Further understanding of the impact of oncometabolites on immune cell functionality will be essential for optimizing targeted immunotherapies [28].

#### 4.1.4 Targeting Histone Lactylation

Chen et al. [133] revealed that MRE11, a key player in HR, undergoes lactylation at K673 by the acetyltransferase CBP in response to DNA damage and ATM phosphorylation. This lactylation enhances MRE11's DNA‐binding affinity, promoting DNA end resection and facilitating HR. Inhibition of CBP or LDH reduces MRE11 lactylation, impairs HR, and increases chemosensitivity in patient‐derived xenograft and organoid models. A cell‐penetrating peptide targeting MRE11 lactylation was shown to inhibit HR, sensitizing cancer cells to cisplatin and PARP inhibitors (PARPi). These results highlight lactylation as a vital regulator of HR, suggesting that the Warburg effect may promote chemoresistance through enhanced HR, offering MRE11 lactylation as a potential therapeutic target. Zong et al. [56] further demonstrated that β‐alanine disrupts lactate binding to AARS1, reducing p53 lactylation and suppressing tumorigenesis in animal models. Similarly, Zhang et al. [134] found that lactate accumulation triggers site‐specific histone lactylation (H3K18la and H4K12la) via a p300/HDAC1/HDAC3‐dependent mechanism, which upregulates ribosomal protein S6 kinases 2 (S6K2) in virus‐infected hosts, thereby facilitating viral infection. These findings suggest that virus‐induced histone lactylation plays a role in virus–host interactions, identifying new potential targets for controlling viral infections [134]. Moreover, Wu et al. [135] identified a histone lactylation‐driven mechanism that induces ferroptosis through METTL3‐mediated m6A modification, indicating that targeting METTL3 could be a promising therapeutic strategy for sepsis‐associated acute lung injury (ALI). De Leo et al. [136] demonstrated that glucose‐driven histone lactylation in monocyte‐derived macrophages upregulates IL‐10 expression and promotes immunosuppressive activity in GBM, through regulation by the PERK–ATF4–GLUT1 axis, highlighting PERK‐driven lactylation as a potential therapeutic target.

#### 4.1.5 HDAC Inhibitors

Moreno‐Yruela et al. [65] discovered that HDAC1‐3 and SIRT1‐3 function as delactylases, with HDAC1‐3 exhibiting significant activity in removing K(L‐la), K(D‐la), and other short‐chain acyl modifications. This finding reveals new roles for class I HDACs and validates pharmacologically targetable enzymes that regulate histone K(L‐la). These insights provide a foundation for further studies into the functional implications of this modification and its regulatory mechanisms. Several HDACi have been developed and are undergoing clinical trials, with some already in use for cancer treatment, such as MS‐275 for uveal melanoma (UVM) [137] and MGCD0103 for liver cancer [138] and T‐cell lymphoma [139].

The therapeutic strategies outlined above have led to the development of multiple agents targeting lactate metabolism and histone lactylation. Representative compounds are summarized in Table 2.

**TABLE 2:** Onco‐therapeutic approaches targeting the lactate axis.

Target	Drug	Research status	Example cancer	Mechanism	Ref/Trial No.
MCT1/2	AZD3965	Clinical trial	Hematopoietic cancer and advanced cancers	Lactate excretion	[130, 140, 141, 142, 143, 144, 145, 146]/NCT01791595
MCT1/2	SR13801	Pre‐clinical	Breast cancers and B‐cell lymphoma	Lactate anabolism	[144, 147]
MCT1/4	7ACCs	Pre‐clinical	Cervix cancer, breast cancer, and CRC	Lactate excretion	[148]
MCT1/4	3‐BrPA	Pre‐clinical	Cervical cancer, bladder cancer, and CRC	Lactate anabolism	[149]
MCT1	BAY8002	Pre‐clinical	Hematopoietic cancer and some solid cancers	Lactate excretion	[150]
MCT1	SR13800	Pre‐clinical	Burkitt lymphoma cell, neuroblastoma, and breast cancer	Lactate excretion	[151, 152]
MCT4	Syrosingopine	Pre‐clinical	Lung cancer, HCC, and breast cancer	Lactate excretion	[141]
MCT4	AZ93	Pre‐clinical	CRC melanoma	Lactate excretion	[153]
MCT4	ALK‐04	Pre‐clinical	Melanoma	Lactate excretion	[154]
MCT	CHC (α‐cyano‐4‐hydroxycinnamate)	Pre‐clinical	Breast cancers	Lactate anabolism	[143, 155]
GPR1	LRH7‐G5	Pre‐clinical	Triple negative breast cancer	Lactate excretion	[150]
HK2	2DG	Clinical trial	Breast cancer, prostate cancer, ovarian cancer, lung cancer, glioma, pancreatic cancer, and osteosarcoma	Glucose uptake	[156]/NCT00633087 and NCT00096707
HK2	Benserazide	Pre‐clinical	Cervical cancer, TSCC, and CRC	Glucose uptake	[157, 158]
HK2	Tristetraprolin	Pre‐clinical	Breast cancer	Glucose uptake	[159]
HK2	BAG3	Pre‐clinical	Pancreatic cancer	Glucose uptake	[160]
HK2	Benitrobenrazide	Pre‐clinical	Pancreatic cancer and CRC	Glucose uptake	[161]
HK2	Ikarugamycin	Pre‐clinical	Pancreatic cancer	Glucose uptake	[162]
PDK	Dichloroacetate	Clinical trial	Brain tumor, GBM, lung cancer, and metastatic breast cancer	Pyruvate catabolism	[163]/ NCT00540176, NCT00566410, NCT01029925, NCT01111097, and NCT01386632
LDHA	AT101	Clinical trial	Pancreatic cancer, breast cancer, adrenocortical carcinoma, SCLC, NSCLC, SCCHN, brain and central nervous system tumors, and hematopoietic cancer	Lactate anabolism	[164, 165]/NCT00275431, NCT00286780, NCT00286793, NCT00286806, NCT00390403, NCT00397293, NCT00440388, NCT00544960, NCT00571675, NCT1285635, and NCT01003769
LDHA	Oxamate	Pre‐clinical	NSCLC, melanoma	Lactate anabolism	[166, 167]
LDHA	Zinc	Clinical trial	Melanoma	Lactate anabolism	[168]/NCT02101008
LDHA	NHI‐Glc‐2	Pre‐clinical	NSCLC and gastric cancer	Lactate anabolism	[169]
LDHA	LncRNA GLTC	Pre‐clinical	PTC	Lactate anabolism	[170]
LDHA/B	NCI‐006	Pre‐clinical	Pancreatic cancer	Lactate anabolism	[171]
LDHA/B	MS6105	Pre‐clinical	Pancreatic cancer	Lactate anabolism	[172]
Lactate	LOx	Pre‐clinical	Breast cancer, melanoma, GBM, and HCC	Lactate catabolism	[173, 174, 175, 176, 177, 178, 179, 180, 181, 182, 183]
Lactate	NaHCO_3_	Pre‐clinical	NSCLC and breast cancer	Lactate catabolism	[184]
Lactate	nanoCaCO_3_	Pre‐clinical	Breast cancer	Lactate catabolism	[185]
Lactate	CoMnFe‐LDO	Pre‐clinical	UM	Lactate catabolism	[186]
GLUT1	BAY876	Pre‐clinical	Breast cancer and GBM	Lactate anabolism	[187, 188]
Pyruvate	UK5099. POx	Pre‐clinical	Breast cancer	Pyruvate catabolism	[189]
Lactylation	BML	Pre‐clinical	HCC	Histone modification	[99]
Lactylation	RJA	Pre‐clinical	HCC	Histone modification	[190]
Lactylation	K673‐pe	Pre‐clinical	CRC	Non‐histone modification	[133]
Lactylation	D34‐919	Pre‐clinical	GBM	Non‐histone modification	[110, 191]
Lactylation	Anti lac‐APOC2‐k70 antibody	Pre‐clinical	NSCLC	Non‐histone modification	[192]
Lactylation	β‐Alanine	Pre‐clinical	CRC	Non‐histone modification	[56]
Lactylation	MG149	Pre‐clinical	CRC	Non‐histone modification	[105]

Abbreviations: CRC, colorectal cancer; GBM, glioblastoma; GLUT1, glucose transporter 1; GPR1, G protein‐coupled receptor 1; HCC, hepatocellular carcinoma; HK2, hexokinase 2; LDHA, lactate dehydrogenase A; LDHB, lactate dehydrogenase B; MCT, monocarboxylate transporters; MCT1, monocarboxylate transporter 1; MCT2, monocarboxylate transporter 2; MCT4, monocarboxylate transporter 4; NSCLC, non‐small cell lung cancer; PTC, papillary thyroid cancer; SCCHN, squamous cell carcinoma of the head and neck; SCLC, small cell lung cancer; TSCC, tongue squamous cell carcinoma; UM, uveal melanoma.

### 4.2 Combined Therapy

Given the multifaceted roles of lactate in immune suppression and metabolic reprogramming, combining lactate‐targeted interventions with immune checkpoint blockade (ICB) offers a promising synergistic strategy. Recent studies highlight how modulation of lactate metabolism or transport can enhance the efficacy of ICB therapies.

#### 4.2.1 Combining Metabolic Modulation With Immune Checkpoint Blockade

The combination of LDH inhibitors with anti‐PD‐1 therapy has demonstrated enhanced anti‐tumor effects. In mouse melanoma models, blocking LDHA has been shown to increase the production of IFN‐γ and granzyme B by CD8^+^ T cells and NK cells, thereby strengthening the immune response to PD‐1 checkpoint inhibitors [128]. Metformin, initially introduced in the late 1950s for the treatment of type 2 diabetes, remains a preferred drug for improving insulin sensitivity as of 2022. Preclinical and clinical research indicates that metformin also possesses antibacterial, antiviral, and antimalarial properties, in addition to its potential for treating various diseases. Furthermore, metformin may help prevent neurodegenerative conditions by directly targeting neural stem cells, inhibiting pro‐inflammatory pathways, and safeguarding mitochondrial and vascular functions [28]. Zhou et al. [193] revealed that metformin suppresses histone H3K18 lactylation, leading to reduced reactive oxygen species (ROS) levels and decreased neutrophil recruitment. The combination of metformin and anti‐PD‐1 therapy has also been found to enhance T‐cell activity and tumor eradication in melanoma mouse models. Likewise, patients with non‐small cell lung cancer receiving both metformin and ICIs exhibit improved response rates and extended overall survival. In patients with HCC responding to anti‐PD‐1 treatment, MOESIN protein lactylation levels in Treg cells are notably lower compared to non‐responders.

#### 4.2.2 Combining Lactate Transport Inhibition With Immune Checkpoint Blockade

Preclinical evidence suggests that combining the MCT1 inhibitor AZD3965 with anti‐PD‐1 therapy reduces lactate secretion into the TME in solid tumors and decreases the presence of PD‐1^+^ Tim‐3^+^ T cells, thereby enhancing anti‐tumor immunity [194]. Moreover, knocking out MCT1 specifically in Tregs has been shown to inhibit tumor growth and can be effectively combined with anti‐PD‐1 therapy, indicating that MCT1 inhibition could play a dual role in curbing tumor progression [76].

Together, these findings underscore the translational potential of combining metabolic and immunologic interventions. Lactate‐targeted strategies not only restore T‐cell activity but also reshape the TME to favor immune clearance, paving the way for more effective and durable cancer immunotherapies.

## 5 Conclusion and Future Directions

Since the discovery of histone lactylation in 2019, the field has rapidly expanded beyond its initial epigenetic implications, unveiling critical roles in cancer biology. This paradigm‐shifting modification has opened a new frontier in the study of how metabolites can directly regulate gene expression and protein function. However, despite the progress, key mechanistic and translational questions remain. These include the full landscape of enzymes responsible for adding, removing, and recognizing lactyl marks; the biological distinctions between l‐ and d‐lactylation, and the potential for therapeutic intervention. The following sections outline several promising directions to guide future research and clinical innovation.

### 5.1 Identifying Additional Enzymes Involved in Histone Lactylation

Since Zhao Yingming's team first identified lactylation modifications in 2019, extensive research has highlighted their critical role in malignant tumors. However, the full scope of the “writers,” “erasers,” and “readers” involved in lactylation remains unclear, and the precise mechanisms by which lactylation regulates physiological functions are still under investigation. The diversity of modification sites means that lactylation occurs not only on histones but also on a wide array of lysine residues in non‐histone proteins. This randomness and complexity make it challenging to fully understand the regulation and functional implications of lactylation. With the advancement of high‐throughput technologies, more enzymes responsible for lactylation modifications are expected to be identified, potentially revealing the underlying mechanisms of this modification. Future studies should test whether novel lysine lactyltranferases beyond p300 and TIP60 act as lactylation “writers.” It is also worth exploring whether “readers” of other acylations (e.g., YEATS and bromodomain) can selectively recognize lactylated lysine residues.

### 5.2 Exploring the Vast Research Landscape of d‐Lactylation Modification

In July 2024, Professor Zhao Yingming's team discovered that both enantiomers of lactate, l‐lactate and d‐lactate, mediate protein modification through l‐lactylation and d‐lactylation, respectively. This discovery significantly broadened the understanding of lactate's biological functions [64]. It raises compelling questions about the distinct roles these two configurations of lactate—and their associated lactylation modifications—play in cellular processes. Could they, like “a song of ice and fire,” regulate metabolic pathways and biological networks in contrasting ways? Many unknowns remain to be explored.

Recent studies have revealed that accumulated l‐lactate in liver cancer stem cells (LCSCs) mediates the l‐lactylation of histones (primarily H4K56) and non‐histone proteins, such as the key glycolytic enzyme ALDOA, thereby regulating the proliferation, migration, and stemness of LCSCs [195]. Similarly, another study found that d‐lactate, a small molecule metabolite produced by gut microbiota, functions as an endogenous immunomodulator, promoting the polarization of TAMs from the M2 phenotype to the M1 phenotype, thereby reshaping the immunosuppressive TME in HCC [196]. These findings provide new insights into the distinct regulatory roles of l‐lactate and d‐lactate in HCC, revealing their broader metabolic functions.

Eukaryotes primarily produce l‐lactate through glycolysis, while prokaryotes mainly generate d‐lactate. Apart from a small amount formed via the methylglyoxal pathway, d‐lactate is also produced by gut microbiota during glucose degradation and is absorbed into the bloodstream via the intestines. Previous studies have shown that d‐lactate, as a gut microbial metabolite, enters the liver through the portal vein and enhances macrophages' capacity to clear blood‐borne pathogens. Recent research further demonstrates that d‐lactate serves as an endogenous immunomodulator, driving the conversion of M2‐type macrophages into M1‐type and reshaping the immunosuppressive TME in HCC.

These two studies have unveiled distinct regulatory mechanisms and functions of l‐lactate and d‐lactate in liver cancer, highlighting their unique roles. To support global profiling of l‐lactylation, Sun et al. [197] developed a bioorthogonal probe, YnLac, which mimics l‐lactate and enables proteome‐wide identification of lactylated sites in mammalian cells, highlighting the technological advantage in l‐lactylation research. This raises the intriguing question of whether, and how, d‐lactate exerts its regulatory influence on cellular processes through d‐lactylation. The mechanisms governing this remain unknown, presenting a vast and untapped area of research.

Clinical studies have linked abnormal d‐lactate metabolism to the development of various diseases, including short bowel syndrome [198], acute neurological injury [199], and HCC [196], among others. These findings suggest that d‐lactylation may play a critical role in the pathology of intestinal, neurological, and oncological diseases. Furthermore, whether there is any crosstalk between d‐lactylation and l‐lactylation in the progression of these conditions is an open question that warrants further exploration.

Exploring this “realm of ice and fire” will open a new chapter in the study of lactate in life sciences and medicine. It promises to deepen our understanding of the complex roles of different lactate forms and their lactylation modifications in the TME, while also offering fresh insights into the diagnosis and treatment of other diseases.

### 5.3 Developing New Diagnostic and Therapeutic Methods

Lactate within the TME regulates transcriptional levels and protein activity via lactylation modifications, significantly influencing malignant tumor progression. This highlights the potential of developing therapeutics that target lactate and lactylation modifications as a viable strategy for cancer treatment. Future research should focus on elucidating the regulatory mechanisms behind lactylation to systematically understand its role in tumorigenesis and other diseases, providing a theoretical foundation for novel tumor‐targeted therapies. A deeper exploration of the biochemical processes involved in lactylation and de‐lactylation is essential. Additionally, the role of lysine lactylation “readers” in modulating transcriptional activity requires further clarification. Investigating the enzymes responsible for producing lactyl‐CoA will illuminate the regulatory processes driving histone lysine lactylation. Zhang et al.’s work has shed light on how lactate influences histone dynamics and gene expression, offering new biological insights. While cancer immunotherapy has gained prominence, current treatments often target immune cell types in isolation. Exploring combination therapies, such as integrating metabolic reprogramming with immune‐based therapies, represents a promising direction for future research. Lactate, once regarded merely as a metabolic byproduct, is now understood to play a critical role in tumor progression by serving as a fuel source, promoting invasion and metastasis, enhancing angiogenesis, and contributing to immune suppression. This reinforces lactate's potential as a key therapeutic target in cancer treatment.

Several important scientific questions remain unresolved. It is yet to be determined whether lactylation can occur on amino acid residues beyond lysine. Additionally, given lactate's broad role within the TME, the full extent of lactate‐mediated lactylation likely surpasses current knowledge. The mechanisms governing lactyl‐CoA synthesis and the function of “writers,” “erasers,” and “readers” that regulate histone lysine lactylation have yet to be thoroughly explored. Furthermore, the interactions between lactylation and other PTMs, such as acetylation and methylation, and their impact on disease prognosis across various pathological conditions remain to be clarified [28].

Numerous approaches targeting metabolic pathways are being developed to enhance the effectiveness of immunotherapy. Given the critical role of lactate and lactylation in tumor metabolic reprogramming and immune regulation, further investigation into protein lactylation's role in modulating anti‐tumor immunity and cancer progression will undoubtedly yield groundbreaking discoveries. To establish causality, it would be valuable to develop in vivo models such as H3K18R knock‐in mice to determine the site‐specific function of histone lactylation in tumorigenesis and immune modulation. These models will be instrumental in validating lactylation as a therapeutic target.

### 5.4 Clinical Relevance and Translational Potential

Recent findings have begun to bridge the gap between lactylation research and clinical application. While the majority of studies to date remain preclinical, emerging human evidence suggests that lactylation may serve as a viable diagnostic and therapeutic axis in cancer and beyond. For example, histone H3K18 lactylation (H3K18la) has been identified as a potential prognostic biomarker in patients with septic shock. Its levels in peripheral blood mononuclear cells correlate positively with inflammatory cytokines and clinical severity scores, indicating that lactylation is not only detectable in human samples but also clinically relevant to disease progression and immune response regulation [46].

Furthermore, lactylation has been implicated in tumor immune evasion through post‐translational [86] modification of immune checkpoint molecules. A recent phase I clinical trial (ChiCTR2300067929) evaluated a serine: glycine‐free diet in patients with advanced solid tumors and demonstrated that this intervention reduces PD‐L1 lactylation and restores T‐cell activity [200]. This study represents the first clinical attempt to modulate lactylation as part of a therapeutic strategy, highlighting the feasibility of targeting the lactate‐lactylation axis in humans.

In addition to these direct insights, upstream regulators of lactylation, particularly lactate production and transport, have entered clinical evaluation. AZD3965, a selective inhibitor of MCT1, has been tested in phase I clinical trials (e.g., NCT01791595) for advanced malignancies [201]. Although promising in preclinical models, case reports of hyperlactatemia‐related toxicity suggest that careful patient stratification and metabolic monitoring are essential [202]. Similarly, CPI‐613 (devimistat), a lipoate analog targeting mitochondrial dehydrogenases in the TCA cycle, has progressed to phase III trials in metastatic pancreatic cancer (NCT03504423) [203] and has been evaluated in early‐phase studies for biliary tract cancer [204] and acute myeloid leukemia [205]. Although the primary endpoints of some studies were not met, these trials collectively demonstrate the translational momentum behind lactate‐targeted therapies.

Taken together, these developments underscore lactylation's growing clinical relevance. Future research should focus on validating lactylation‐based biomarkers in cancer patient cohorts, exploring the therapeutic value of modulating lactylation in combination with immunotherapy or metabolic inhibitors, and establishing the safety and efficacy of lactate‐modulating agents in precision oncology. These efforts will be essential to transform lactylation from a mechanistic insight into a clinically actionable pathway.

In summary, the rediscovery of lactate as a central regulator in tumor biology and the identification of protein lactylation as a novel PTM have opened a new chapter in cancer research and beyond. While significant progress has been made in deciphering the metabolic and epigenetic roles of lactate, many mechanistic questions remain unresolved, and clinical translation is still in its infancy. Future research that systematically explores the enzymatic machinery of lactylation, its functional heterogeneity across disease contexts, and its interactions with other metabolic and epigenetic pathways will be critical. As tools and models advance, the integration of lactylation‐targeted strategies with immunotherapy and metabolic modulation holds great promise. Lactate, once seen as a waste product, is now at the forefront of a paradigm shift—reshaping our understanding of disease and redefining therapeutic possibilities.

## Author Contributions

L.Z. and Z.S. proposed this theme, organized the structure, provided the raw materials, and selected the cited references. L.Z., H.C., and Y.L. reorganized the figures and wrote the manuscript. Y.Y. and Z.S. revised the manuscript and oversaw the project. All authors reviewed the manuscript. All authors have read and approved the final version.

## Ethics Statement

The authors have nothing to report.

## Conflicts of Interest

The authors declare no conflicts of interest.

## Data Availability

All data needed to evaluate the conclusions described in the paper are presented in the paper and/or in the Supporting Information. Additional data related to this paper can be requested from the corresponding authors.
